# Regulation by Trace Amine-Associated Receptor 1 (TAAR1) of Dopaminergic-GABAergic Interaction in the Striatum: Effects of the Enhancer Drug (-)BPAP

**DOI:** 10.1007/s11064-025-04337-7

**Published:** 2025-02-04

**Authors:** Laszlo G. Harsing, Gábor Szénási, Balázs Fehér, Ildikó Miklya

**Affiliations:** 1 Department of Pharmacology and Pharmacotherapy, Semmelweis University, Budapest, Hungary; 2 Center for Pharmacology and Drug Research & Development, Semmelweis University, Budapest, Hungary; 3 Institute of Translational Medicine, Semmelweis University, Budapest, Hungary; 4 Budapest University of Technology and Economics, Budapest, Hungary

**Keywords:** (-)BPAP, Dopaminergic activity enhancer effect, Trace amines / Trace amine-associated receptor 1, [^3^H]Dopamine and [^3^H]GABA release, Rat striatum

## Abstract

Although it is well documented that the striatal GABAergic projection neurons receive excitatory and inhibitory dopaminergic innervation via D1 and D2 receptors, the trace amine-associated receptor 1 (TAAR1)-mediated regulation of this neural connection is much less studied. The presence of TAAR1 was originally detected in brain aminergic neurons, with recent evidence indicating its presence in striatal GABAergic neurons as well. The objective of the present study was to demonstrate the role of TAAR1 and signaling in dopaminergic-GABAergic interaction in the neural circuitry of the striatum. Besides trace amines, which are considered natural ligands for TAAR1, series of different exogenous drugs were identified to act on this receptor. Using the dopaminergic activity enhancer compound (-)BPAP ((-)-1-(benzofuran-2-yl)-2-propylaminopentane HCl), a potential agonist for TAAR1, we have found that it increased the electrical stimulation-induced [^3^H]dopamine release in rat striatal slices. This effect of (-)BPAP occurred parallel with increases of [^3^H]GABA release in striatum when used in 10^–13^–10^–11^ mol/L concentrations. The effects of (-)BPAP on the release of both neurotransmitters were bell-shaped. We speculated that the rising phase of the concentration-effect curves was evoked by an agonist effect of (-)BPAP on TAAR1 whereas the declining phase was a result of heterodimerization of TAAR1 with pre- and postsynaptic dopamine D2 receptors. The bell-shaped curves suggest that the (-)BPAP-induced heterodimerization of TAAR1 with dopamine D2 receptors may switch off TAAR1 signaling and switch on transduction coupled to D2 receptors. We also suggest that (-)BPAP increases synaptic strength in a hypothetical quadrilateral neuronal organization consisting of dopaminergic nerve ending, GABAergic neurons, trace amine-producing D cells, and supportive glial cell processes.

## Introduction

Knoll and Coworkers [1] described a series of aryl-2-propylaminopentane molecules, which increased the electrical stimulation-induced catechol- and indolamine release from brain tissues without affected resting neurotransmitter release. These compounds are designated as catecholaminergic activity enhancer (CAE) drugs. Enhancer drugs increased depolarization-mediated noradrenaline, dopamine, and serotonin release in vitro in unusually low, pico/nano molar concentrations, effective concentrations varied between 10^–13^ and 10^–9^ mol/L [2]. The enhancer effect on neurotransmitter release was demonstrated in slices containing the substantia nigra, locus coeruleus, and the raphe nuclei, areas in the mesencephalon containing cell bodies of biogenic amine projection neurons. Subsequent studies also demonstrated that the dopamine releasing effect of the enhancer compounds is present not only in neural cell bodies but also in the regions of these neurons containing axon terminals. Thus, the enhancer effect was demonstrated in the prefrontal cortex, olfactory bulb, and the striatum as well [3]. Although a series of enhancer molecules had been synthesized, (-)PPAP, (-)IPAP, and (-)BPAP, aryl-2-propylaminopentane compounds containing phenyl, indole, and benzofuran rings, respectively, were selected for detailed investigations both in vitro and in vivo [4]. Interesting enough, the chemically related selegiline (L-deprenyl), a selective inhibitor of B type monoamine oxidase and antiparkinsonian drug, also possesses enhancer activity in dopaminergic neurotransmission [5, 6].

Almost parallel in time, two laboratories described independently a specific binding site for the trace amines phenylethylamine, tyramine, and tryptamine [7, 8]. This binding site was designated as trace amine-associated receptor (TAAR) and appeared in several different isoforms (TAAR1-7). TAAR1 is probably the most widely studied in terms of their roles in the central nervous system in health and disease [9–13]. The presence of TAAR1 was demonstrated in brain nuclei containing noradrenergic, dopaminergic, and serotonergic neurons and in projection brain areas such as the cerebral cortex, olfactorial bulb, and caudate nucleus [14, 15]. TAAR1 may have a key role in regulation of events in dopaminergic axon terminals, like transporter operation, autoreceptor-mediated feedback inhibition, neurotransmitter release with cytoplasmic and vesicular origins [16, 17]. Both agonist (RO5166017, RO5263397) and antagonist (EPPTB) compounds for TAAR1 have been synthesized and extensively studied [18, 19].

**Fig. 1:**
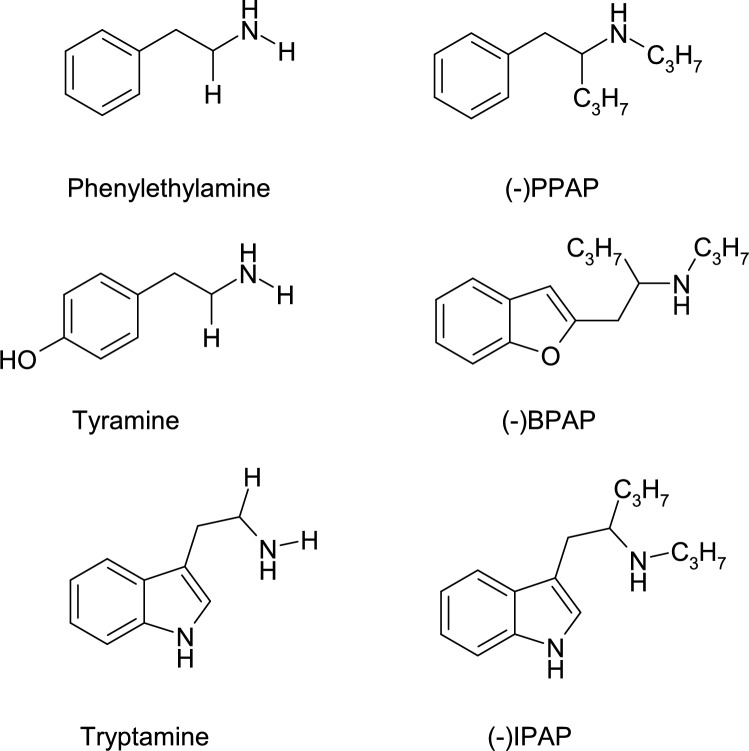
The chemical structures of the trace amines (phenylethylamine, tyramine, tryptamine) and catecholamine activity enhancer aryl-2-propylaminopentane drugs (-)PPAP, (-)BPAP, (-)IPAP) [20]

Comparison of the enhancer and TAAR1 regulations led us to recognize several similarities. These similarities include: 1. The chemical structures of the enhancer drugs and trace amines (Fig. 1); 2. The site of action of enhancer drugs and trace amines in brain nuclei containing cell bodies of aminergic neurons and their axon terminals; 3. Pharmacological actions of the enhancer drugs and trace amines on neurotransmitter release appearing in unusually low pico/nano molar concentrations; 4. The abilities of the enhancer drugs and trace amines to strengthen the efficacy of catechol- and indolaminergic neural activities in the central nervous system. It is, however, a basic difference between the mechanism of action of enhancer ligands and trace amines that the former drugs are without effect on resting neurotransmitter release, whereas the latter ones evoke mark increases [17].

Concerning these similarities, we have speculated that the site of action of the enhancer drugs might be the TAAR1 [3]: indeed, we were able to prove this concept by using the TAAR1 selective antagonist EPPTB [21] in enhancer research [6, 17]. Our finding also revealed a possible link between the catecholaminergic enhancer regulation and operation of TAAR1 on neurochemical transmission in the brain.

With further accumulation of knowledge on trace amines and their receptors, evidence was found that TAAR1 is present not only in aminergic neurons, but it may also be expressed in amino acid transmission [22, 23]. In the striatum, the classical amine dopamine possesses receptors and out of these, dopamine D2 receptors bear distribution similar to that of TAAR1: they are expressed both in dopaminergic nerve endings and postsynaptic GABAergic neurons [24]. The aim of the present study was to search for interactions between the receptors of trace amines and that of the classical amine dopamine in striatal neurotransmission. It was concluded that the enhancer drug (-)BPAP, depending on its concentrations used, is able to exert both excitatory and inhibitory influences on this interaction.

## Materials and Methods

### Ethical Considerations

All experimental procedures were approved by local Ethical Committees and were in accordance with the NIH Guide for the Care and Use of Laboratory Animals, 8th Edition, 2011. Animal care and handling protocols were approved by the regional animal health authority in Hungary (Pest County Government Office, resolution number: PE/EA/285–5/2020, date: March 19, 2020).

### Animals

Male Wistar rats weighing 180–200 g were purchased from Toxicoop Ltd., Budapest, Hungary and used for investigations. The animals were housed five to a cage in a temperature- and humidity-controlled animal facility on a 12-h light/dark cycle (6.00 a.m. on, 6.00 p.m. off) with food and water available ad libitum. All efforts were done to minimize the harm to animals.

### Preparation of Rat Brain Slices

Rats were decapitated by guillotine under light ether anesthesia and the brains were removed from the skull. The striatum was dissected according to Glowinski and Iversen [25] and slices were prepared by a McIlwain tissue chopper (Ted Pella Inc., Redding, CA, USA). The brain slices were collected and immersed in Krebs-bicarbonate buffer aerated with 95% O_2_/5% CO_2_ gas mixture (pH 7.4) at room temperature. The composition of the buffer was in mmol/L: NaCl 118, KCl 4.7, CaCl_2_ 1.25, NaH_2_PO_4_ 1.2, MgCl_2_ 1.2, NaHCO_3_ 25, glucose 11.5, ascorbic acid 0.3, and Na_2_EDTA 0.03).

### Release of [^3^H]Dopamine from Rat Brain Slices

Striatal slices from rat brain were loaded with [^3^H]dopamine (10 µCi) for 30 min in 1.5 ml oxygenated and preheated (37 °C) Krebs-bicarbonate buffer [26]. After loading the tissues with [^3^H]dopamine, the slices were transferred into low volume (0.3 ml) superfusion chambers (Experimetria Kft, Budapest, Hungary) and superfused with aerated and preheated Krebs-bicarbonate buffer. The flow rate was kept at 1 ml/min by a Gilson multichannel peristaltic pump (type M312, Villiers-Le Bel, France). The superfusate was discarded for the first 60 min period of the experiments, then twenty-five 3-min fractions were collected by a Gilson multichannel fraction collector (type FC-203B, Middletown, WI, USA). When used, biphasic electrical field stimuli (40 V voltage, 10 Hz frequency, 2-ms impulse duration for 3 min) were delivered in fractions 4 and 18 by electrostimulators (Grass S88 Stimulator, Quincy, MA, USA) to evoke increase of [^3^H]dopamine release. If otherwise not indicated, drugs were added to brain slices from collected fraction 8 and maintained throughout the experiments.

### Release of [^3^H]GABA from Rat Brain Slices

Striatal slices from rat brain were loaded with [^3^H]GABA (5 µCi) for 30 min in 1.5 ml aerated and preheated (37 °C) Krebs-bicarbonate buffer containing 1 mmol/L β-alanine [27]. After loading the tissues with [^3^H]GABA, the brain slices were transferred into superfusion chambers and superfused as described above. The buffer contained (aminooxy)acetic acid and nipecotic acid in concentrations of 0.1 mmol/L. The flow rate was kept at 1 ml/min by a Gilson multichannel peristaltic pump. The superfusate was discarded for the first 60 min period of the experiments, then twenty-five 3-min fractions were collected by a Gilson multichannel fraction collector. When used, biphasic electrical field stimuli (40 V voltage, 20 Hz frequency, 2-ms impulse duration for 6 min) were delivered in fractions 15 and 16 by electrostimulators to evoke [^3^H]GABA efflux. If otherwise not indicated, drugs were added to striatal slices from fraction 5 and maintained throughout the experiments.

### Determination of [^3^H]Dopamine and [^3^H]GABA Efflux

At the end of the experiment, tissues were collected from the superfusion chambers, weighed and solubilized in 0.4 ml Soluene-350. An aliquot (50 µl) was mixed with 5 ml of liquid scintillation reagent and subjected to liquid scintillation spectrometry for determination of tissue content of radioactivity. The tissue content of [^3^H]dopamine or [^3^H]GABA was expressed as kBq/g tissue.

To determine the radioactivity released from brain slices, a sample (1 ml) of the superfusate was mixed with 5 ml of liquid scintillation reagent and subjected to liquid scintillation spectrometry. The efflux of [^3^H]dopamine or [^3^H]GABA was expressed in kBq/g/3 min fraction or as a fractional rate, i.e. a percentage of the amount of radioactivity in the tissue at the time of the release was determined. To estimate the electrically induced [^3^H]dopamine or [^3^H]GABA overflow, the mean of the basal efflux determined before and after stimulation was subtracted from each sample and summed [26].

The effect of drugs on resting [^3^H]dopamine release was determined in the presence and absence of drugs and was expressed by the ratio of [^3^H]dopamine efflux in fractions 17 and 3 (basal outflow, B2/B1). The effect of drugs on electrically stimulated [^3^H]dopamine release was expressed by the ratio of [^3^H]dopamine efflux determined in response to the 2nd (fraction 18, presence of drug) and 1st (fraction 4, absence of drug) stimulations (S2/S1).

The effect of drugs on resting [^3^H]GABA release was determined in the presence and absence of drugs and was expressed by the ratio of [^3^H]GABA efflux in fractions 14 and 4 (basal outflow, B2/B1). The effect of drugs on electrically stimulated [^3^H]GABA release was expressed as a fractional rate and the release determined in drug-treated and drug-free (control) experiments were compared. The Quattro Pro and the GraphPad Prism computer programs were used for data calculation.

### Statistical Analyses

The Student *t*-Statistics for two-means, and one-way ANOVA followed by the Dunnett’s test were used for statistical analysis of the data as appropriate. In [^3^H]GABA release experiments, one-way ANOVA followed by the Dunnett’s test was carried out following logarithmic transformation of the data. The mean ± S.E.M. was calculated and the number of independent determinations was indicated with n. A level of probability (p) less than 5% was considered significant.

### Chemicals Used in this Study

[^3^H]Dopamine (dihydroxyphenylethylamine-3,4[^3^H], specific activity: 27.8 Ci/mmol), [^3^H]GABA (aminobutyric acid, γ-[2,3-^3^H (N)]), specific activity: 26 Ci/mmol), Soluene-350, and Ultima Gold XR liquid scintillation reagent were obtained from Per-Form Hungária Kft, PerkinElmer Life and Analytical Sciences, Boston, MA, USA). β-Alanine, (aminooxy)acetic acid, nipecotic acid, (S)-(-)-sulpiride, and EPPTB (Ro5212773, N-(3-ethoxyphenyl)−4-pyrrolidin-1-yl-3-trifluoromethylbenzamide) were purchased from Sigma-Aldrich Chemical Co, Budapest, Hungary. (-)BPAP ((-)1-(benzofuran-2-yl)−2-propylaminopentane HCl) was received from Fujimoto Pharmaceutical Corp., Osaka, Japan. All other chemicals were of analytical grade.

## Results

### Resting and Electrical Stimulation-Induced [^3^H]Dopamine Release from Rat Striatum: Effect of (-)BPAP

**Fig. 2:**
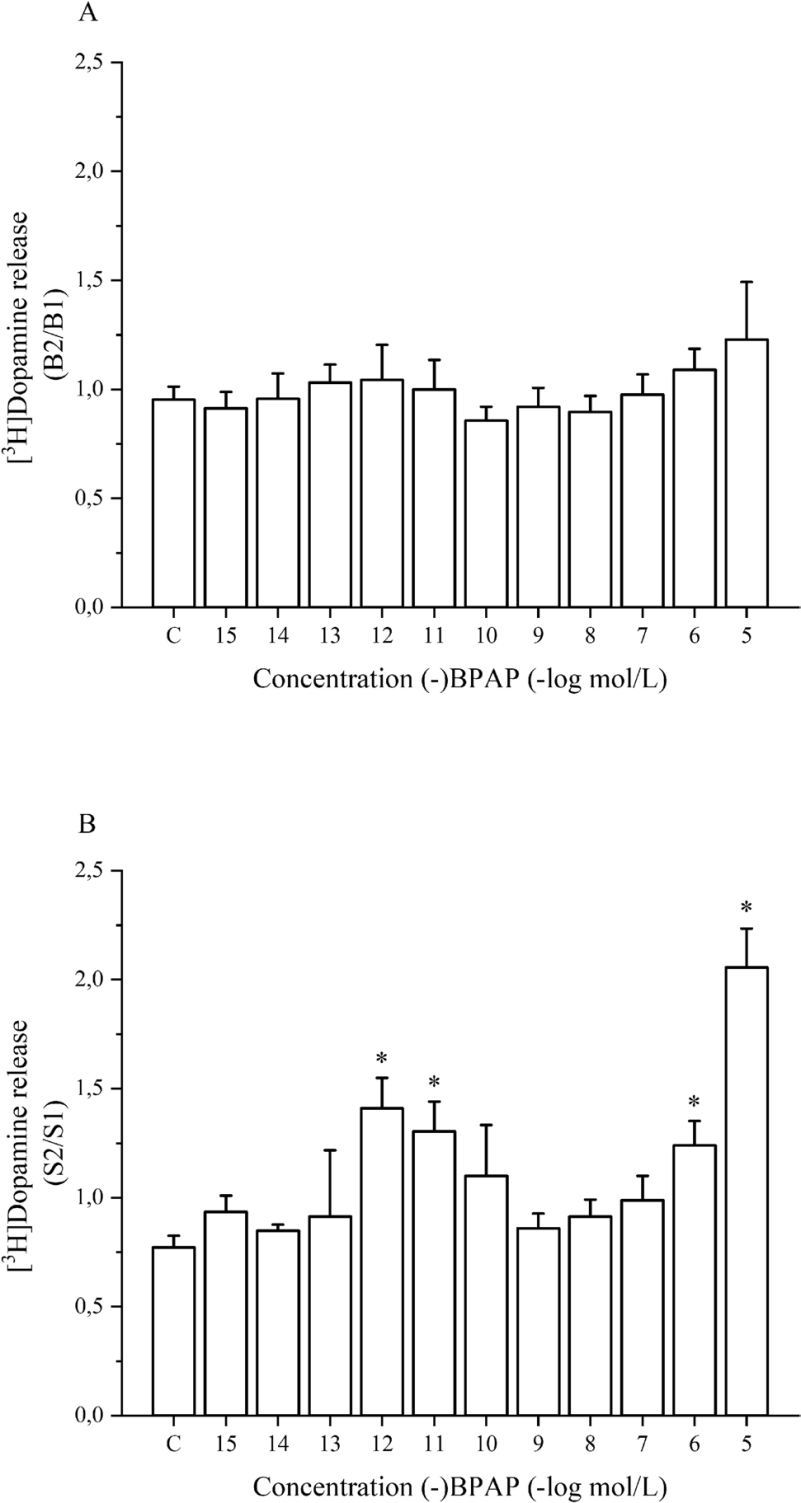
Concentration-dependent effects of (-)BPAP on resting and electrical stimulation-induced [^3^H]dopamine release from rat striatum. The resting and electrical stimulation-induced [^3^H]dopamine release was determined as a fractional rate. (-)BPAP was added to the superfusion buffer from fraction 8 in a concentration range from 10^–15^ to 10^–5^ mol/L. **A**: Resting [^3^H]dopamine release was determined in fractions 3 (basal outflow in the absence of drug, B1) and 17 (basal outflow in the presence of drug, B2) and the B2/B1 ratio was calculated. The B2/B1 value was 0.95 ± 0.05 (n = 8) in control **c** experiments. (-)BPAP added in these concentrations was without effect on resting [^3^H]dopamine release. One-way ANOVA followed by the Dunnett’s test, F(11,40) = 0.464, p = 0.914, mean ± S.E.M., n = 3–8. **B**: The effect of (-)BPAP on electrical stimulation-induced [^3^H]dopamine release. Electrical stimulation (40 V, 10 Hz, 2-ms for 3 min) was applied in the 1st (absence of drug, S1) and 2nd (presence of drug, S2), stimulations carried out in fractions 4 and 18 and the release was expressed as S2/S1 ratio. The S2/S1 value was 0.77 ± 0.05 (n = 8) in control experiments. Note: (-)BPAP exerted a dual-effect: it increased electrical stimulation-induced [^3^H]dopamine release in 10^–12^, 10^–11^ and 10^–6^, 10^–5^ mol/L concentrations. One-way ANOVA followed by the Dunnett’s test, F(11,40) = 7.743, p = 0.0001, *p < 0.05, mean ± S.E.M., n = 3–8

To determine whether (-)BPAP exerts dopaminergic activity enhancer effect, the drug was added to striatal slices in increasing concentrations. As shown in Fig. 2A, (-)BPAP added to superfused striatal slices in a concentration range of 10^–15^ to 10^–5^ mol/L was without effect on resting [^3^H]dopamine release.

In contrast, (-)BPAP increased the electrical stimulation-induced [^3^H]dopamine release from the striatum when added in a concentration range of 10^–15^ to 10^–5^ mol/L. This effect of (-)BPAP was biphasic: it increased electrical stimulation-induced release of [^3^H]dopamine in concentrations of 10^–12^ and 10^–11^ mol/L and 10^–6^ and 10^–5^ mol/L, respectively (Fig. 2B). The former effect was considered as a specific dopaminergic activity enhancer effect. This finding suggests that (-)BPAP primarily alters neuronal activity-dependent dopamine release and it possesses only marginal effect on external Ca^2+^-independent resting neurotransmitter release: these are characteristics for enhancer compounds.

### EPPTB Reversed the Enhancer Effect of (-)BPAP on [^3^H]Dopamine Release in Rat Striatum

Data shown in Table 1 indicate that EPPTB, the first described selective antagonist of TAAR1, suspended the enhancer effect of (-)BPAP on striatal [^3^H]dopamine release. When this interaction was studied, (-)BPAP was used in a concentration of 10^–12^ mol/L in the presence and absence of 10^–7^ mol/L EPPTB, a specific concentration for TAAR1 inhibition.

**Table 1:** EPPTB reversed the enhancer effect of (-)BPAP on electrical stimulation-induced [^3^H]dopamine release in rat striatal slices

Compounds	Concentration (mol/L)	[^3^H]dopamine release (S2/S1)	Significance (p)
1. Control	-	0.83 ± 0.04	
2. (-)BPAP	10^–12^	1.58 ± 0.04*	1:2 < 0.001
3. EPPTB	10^–7^	0.92 ± 0.11	1:3 N.S
4. (-)BPAP +	10^–12^ +		
EPPTB	10^–7^	0.99 ± 0.10	2:4 < 0.0
			3:4 N.S

Reversal by EPPTB of the (-)BPAP-induced [^3^H]dopamine release in rat striatum. For experimental procedure see Fig. 2. The electrical stimulation-induced [^3^H]dopamine release was determined in the 1st (absence of drug, S1) and 2nd (presence of drug, S2), electrical stimulations were carried out in fractions 4 and 18 and the S2/S1 ratio was calculated. (-)BPAP was added to striatal slices from fraction 8 in a concentration of 10^–12^ mol/L and maintained through the experiment in the presence and absence of EPPTB. When used, EPPTB was added to striatal slices from fraction 1 in a concentration of 10^–7^ mol/L and was maintained through the experiment. These drugs did not affect resting [^3^H]dopamine release in rat striatum. One-way ANOVA followed by the Dunnett's test, F(3,12) = 14.47, p = 0.0003, *p < 0.05 vs control. Significance: Student *t*-Statistics for two-means, mean ± S.E.M., n = 4

### Resting and Electrical Stimulation-Induced [^3^H]GABA Release from Rat Striatum: Effect of (-)BPAP

**Fig. 3:**
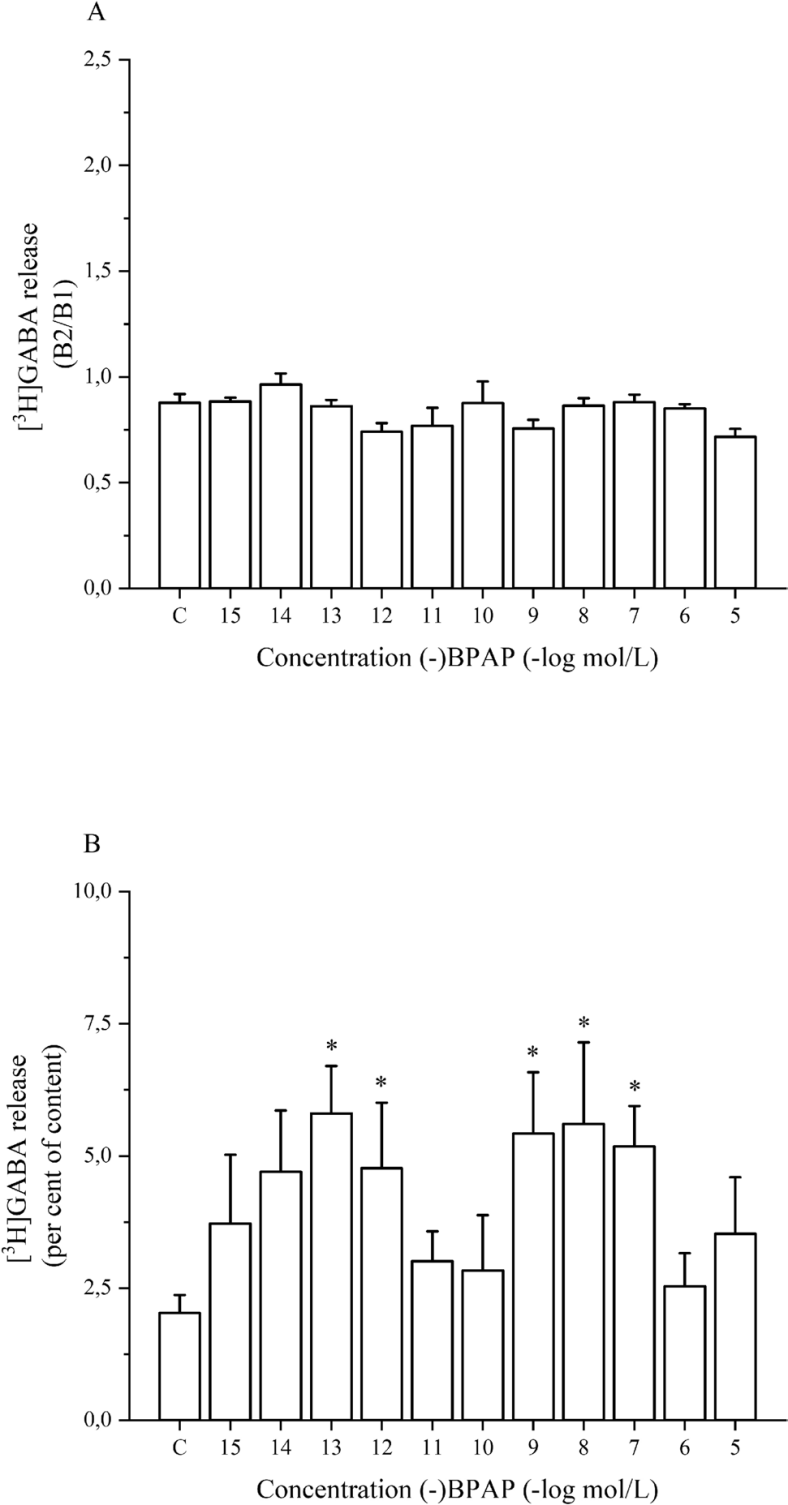
The effect of (-)BPAP on resting and electrical stimulation-induced [^3^H]GABA release from rat striatum. The resting and the electrical stimulation-induced [^3^H]GABA release was determined as a fractional rate. (-)BPAP was added in a concentration range from 10^–15^ to 10^–5^ mol/L to the superfusion buffer from fraction 8 and maintained through the experiment. **A**: The B2/B1 ratio indicates the effect of (-)BPAP on resting [^3^H]GABA release determined in fractions 4 (absence of drug, B1) and 14 (presence of drug, B2). The B2/B1 value was 0.87 ± 0.04 (n = 10) in control experiments (c). One-way ANOVA followed by the Dunnett’s test, F(11,58) = 2.136, p = 0.031, the Dunnett’s test did not indicate significant changes, mean ± S.E.M., n = 4–10. **B**: The effect of (-)BPAP on electrical stimulation-induced [^3^H]GABA release. The stimulation (40 V, 20 Hz, 2-ms for 6 min) was applied in the presence and absence of the drug in collected fractions 15 and 16. Control (c) [^3^H]GABA release was 2.02 ± 0.34 per cent of content released (n = 10). Note: (-)BPAP exerted a dual-effect: it increased electrical stimulation-induced [^3^H]GABA release in 10^–13^ and 10^–12^ and 10^–9^ to 10^–7^ mol/L concentrations. Data in Fig. 3B were subjected to logarithmic transformation and one-way ANOVA followed by the Dunnett’s test, F(11,58) = 2.825, p = 0.0051, *p < 0.05, mean ± S.E.M., n = 4–10

To determine whether (-)BPAP alters [^3^H]GABA release, the drug was added to striatal slices in increasing concentrations. Figure 3A. shows that (-)BPAP was without effect on resting [^3^H]GABA release in rat striatum when added in a concentration range of 10^–15^ to 10^–5^ mol/L.

(-)BPAP added in increasing concentrations from 10^–15^ to 10^–5^ mol/L to superfused striatal slices elicited a biphasic effect on electrical stimulation-induced [^3^H]GABA release: the release was increased in 10^–13^–10^–12^ and 10^–9^–10^–7^ mol/L concentrations (Fig. 3B). The former effect was considered as an enhancer effect appearing on GABAergic neurotransmission. This effect of (-)BPAP on electrical stimulation-induced [^3^H]GABA release showed bi-modal characteristics similar to that observed in depolarization-evoked [^3^H]dopamine release.

### Sulpiride Reversed the Stimulatory Effect of (-)BPAP on [^3^H]GABA Release in Rat Striatum

Data shown in Table 2 indicate that sulpiride, a dopamine D2 receptor antagonist, suspended the enhancer effect of (-)BPAP on striatal [^3^H]GABA release. When this interaction was studied, (-)BPAP was used in a concentration of 10^–8^ mol/L in the presence and absence of 10^–6^ mol/L sulpiride.

**Table 2:** Sulpiride reversed the effect of (-)BPAP on electrical stimulation-induced [^3^H]GABA release in rat striatal slices

Compounds	Concentration (mol/L)	[^3^H]GABA release (per cent of content)	Significance (p)
1. Control	-	3.19 ± 0.32	
2. (-)BPAP	10^–8^	6.13 ± 0.80*	1:2 p < 0.05
3. Sulpiride	10^–6^	4.19 ± 1.19	1:3 N.S
4. (-)BPAP +	10^–8^ +		
Sulpiride	10^–6^	3.35 ± 0.69	2:4 p < 0.05
			3:4 N.S

Reversal by sulpiride of the effect of (-)BPAP on electrical stimulation-induced [^3^H]GABA release in rat striatum. For experimental procedure see Fig. 3. The resting and the electrical stimulation-induced [^3^H]GABA release was determined as a fractional rate. In order to increase [^3^H]GABA release, electrical stimulation (40 V, 20 Hz, 2-ms 6 min) was applied in collected fractions 15 and 16. (-)BPAP was added to striatal slices from fraction 8 ina concentration of 10^–8^ mol/L and maintained through the experiment in the presence and absence of sulpiride. When used, sulpiride was added to striatal slices from fraction 1 ina concentration of 10^–6^ mol/L and was maintained through the experiment. One-way ANOVA followed by the Dunnett's test, F(3,12) = 3.371, p = 0.054, *p < 0.01. Significance: Student *t*-Statistics for two-means, mean ± S.E.M., n = 4

### Comparison of the Effects of (-)BPAP on Resting and Electrical Stimulation-Induced [^3^H]Dopamine and [^3^H]GABA Release from Rat Striatum

**Fig. 4:**
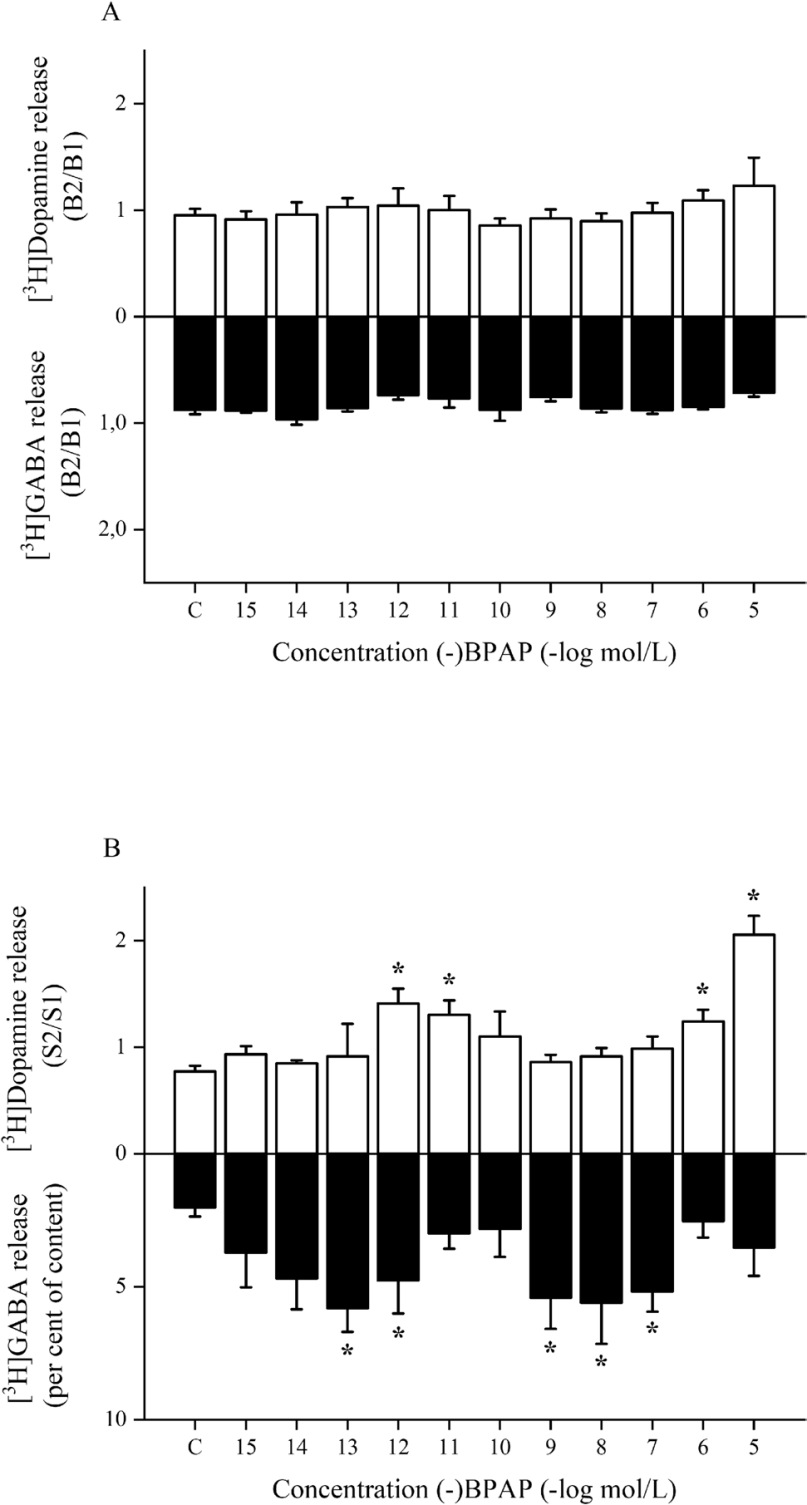
Comparison of the effects of (-)BPAP on resting and electrical stimulation-induced [^3^H]dopamine and [^3^H]GABA release from rat striatum. (-)BPAP was added to the tissue in a concentration range of 10^–15^ to10^−5^ mol/L. Data were collected from Figs. 2 and 3 and plotted repeatedly here for comparison. **A**: The B2/B1 ratios indicate no effects of (-)BPAP on resting [^3^H]dopamine and [^3^H]GABA outflow. **B**: Effects of (-)BPAP on the electrical stimulation-induced [^3^H]dopamine and [^3^H]GABA release. Effect on [^3^H]dopamine release was expressed by the S2/S1 ratios, that on [^3^H]GABA release was expressed as per cent of content released in response to electrical stimulation. For details of determinations and statistical analyses see Figs. 2 and 3, one-way ANOVA followed by the Dunnett’s test, *p < 0.05 indicates significant increases in release when compared to control (**c**), mean ± S.E.M

In Fig. 4A and B, we compared the concentration-effect curves of (-)BPAP on resting and electrical stimulation-induced [^3^H]dopamine and [^3^H]GABA release measured in striatal slices. These concentration-effect curves pointed out that the dopaminergic activity enhancer drug (-)BPAP influences only the electrically stimulated neurotransmitter release but not neurotransmitter efflux that occurs at rest.

Depending on (-)BPAP concentrations used, three different sections can be identified in curves shown in Fig. 4B. Adding (-)BPAP in the enhancer concentrations (10^–13^−10^–11^ mol/L), both [^3^H]dopamine and [^3^H]GABA release increased. These effects were proposed to be an agonist effect of (-)BPAP on TAAR1. Increased concentrations of (-)BPAP altered the release of the two neurotransmitters in opposite directions: 10^–9^−10^–7^ mol/L concentrations of (-)BPAP decreased [^3^H]dopamine release and evoked increases in [^3^H]GABA release. Further, (-)BPAP added in 10^–6^−10^–5^ mol/L concentrations, an increased [^3^H]dopamine release was found that was associated with a declined [^3^H]GABA overflow.

## Discussion

Our main result in this study is that picomolar concentrations of the dopaminergic activity enhancer compound (-)BPAP increases the electrical stimulation-induced [^3^H]dopamine and [^3^H]GABA release in rat striatal slices. The concentration-effect curves of (-)BPAP are bell-shaped and bi-modal and the effects at low concentrations may be presumably due to an agonist effect on TAAR1. Activation of dopaminergic and GABAergic neurons by (-)BPAP occurs simultaneously when used in a concentration range of 10^–13^–10^–11^ mol/L. These findings speak for a novel regulation of the neural circuitry in the striatum.

### Effect of the Enhancer Drug (-)BPAP on Dopaminergic Neurotransmission

We have previously demonstrated that (-)BPAP, a member of the catecholaminergic activity enhancer drugs, stimulated the depolarization-evoked dopamine release in picomolar concentrations and the present data confirm these findings. A characteristic of this drug-effect is that although (-)BPAP increased exocytotic dopamine release, the transporter reversal-mediated dopamine outflow was not affected in resting conditions [2, 17].

We consider a major breakthrough in the enhancer drug research that EPPTB, a selective antagonist of TAAR1 [21], suspended the enhancer effect of (-)BPAP on electrical stimulation-induced [^3^H]dopamine release in rat striatum [17]. TAAR1 is an intracellularly located Gs protein-coupled metabotropic receptor, its signaling increases cAMP production and PKA-mediated phosphorylation [16]. In addition, the downstream of PKA activation results in increases of PKC-mediated phosphorylation [22, 28]. Since PKC is responsible for switching phosphorylated dopamine transporter operation into the reverse-mode [29] as well as phosphorylation of vesicular proteins involved in the exocytosis process [30], we speculated that multiple pools of PKC might be involved in phosphorylation of this carrier and vesicular proteins [31]. Our experiments also suggest that the enhancer drug (-)BPAP, acting as an agonist on TAAR1, activates that pool of PKC, which is responsible for vesicular protein phosphorylation upregulating the readily releasable pool of synaptic dopamine [17, 32].

It has been concluded that presynaptic events, like direction of plasma membrane transporter operation, neurotransmitter release with cytoplasmic origin, and autoreceptor-mediated feedback inhibition are under the control of TAAR1 signaling [15, 16]. Using (-)BPAP, we have demonstrated that vesicular dopamine release is also a TAAR1-controlled process. The possible mechanism of (-)BPAP in the latter event is the PKC-mediated phosphorylation of vesicular proteins involved in exocytosis and VMAT2 that determines accumulation of dopamine in the synaptic vesicles [17, 33]. The biological substrates of these TAAR1-mediated influences are the nigrostriatal dopaminergic axon terminals in the striatum.

The concentration-dopamine release curve of (-)BPAP indicated a second increase in neurotransmitter release, which appeared at micromolar concentrations (10^–6^–10^–5^ mol/L) of the enhancer drug. Mechanisms, which may be involved in this late effect may be a weak inhibition of type A monoamine oxidase (MAO) [34] or a disruption of vesicular pH gradient by protonated (-)BPAP similarly to amphetamine substances [35–37].

### Effect of the Enhancer Drug (-)BPAP on GABAergic Neurotransmission

The nigrostriatal dopaminergic pathway innervates GABAergic medium-sized spiny neurons in the striatum [24]. The closed link between striatal dopaminergic and GABAergic neurons appeared in the TAAR1-mediated enhancer regulation as well. (-)BPAP, added in concentrations of 10^–13^–10^–12^ mol/L, increased the electrical stimulation-evoked [^3^H]GABA release in striatal slice preparations. Since TAAR1 is expressed not only in dopaminergic axon terminals but postsynaptic GABAergic neurons as well [21, 38–40], we assumed that the enhancer effects of (-)BPAP on [^3^H]dopamine and [^3^H]GABA release are two effects develop independently. This assumption is supported by our findings that the enhancer effect of (-)BPAP and the dopamine D1 and D2 receptor-mediated influences on striatal [^3^H]GABA release were observed in different concentration ranges [41].

Increasing the applied concentrations of (-)BPAP to the non-selective enhancer range (10^–10^–10^–5^ mol/L) altered the release of the two neurotransmitters in opposite directions: when (-)BPAP decreased [^3^H]dopamine release [^3^H]GABA release was increased and further, increased [^3^H]dopamine release was associated with opposite changes in [^3^H]GABA release. We concluded that dopamine D2 receptors might be involved in these changes of neurotransmitter release as the dopamine D2 receptor antagonist sulpiride suspended [^3^H]GABA release stimulation evoked by 10^–8^ mol/L concentration of (-)BPAP.

It is worth mentioning that the molecular bases of exocytosis-mediated vesicular GABA release may differ from that of other biogenic amines in external Ca^2+^-dependence or PKC-mediated phosphorylation [42, 43]. Thus, the mechanisms through which the interaction between (-)BPAP and TAAR1 affect biogenic amine and amino acid neurotransmissions may deviate in neural tissues.

### Interaction Between TAAR1 and Dopamine D2 Receptors Plays a Role in the Enhancer Effect of (-)BPAP

In the case of [^3^H]dopamine and [^3^H]GABA release, the concentration-effect curves of (-)BPAP exhibited a bell-shape suggesting that more than one event is involved in this neurochemical effect. Although we explain the rising phase of the concentration-effect curves by increases in readily releasable neurotransmitter pool size, TAAR1 and dopamine D2 receptor heterodimerization [39, 44] can be involved in the declining phase. The decline in the concentration-effect curves occurred when the applied concentrations of (-)BPAP were elevated towards the non-selective enhancer range. In the case of [^3^H]dopamine release, we assume that this decline may be a consequence of an interplay between TAAR1 and presynaptic dopamine D2 autoreceptors in dopaminergic nerve endings [22]. Upon TAAR1-D2 receptor heterodimerization, TAAR1 function may decrease, whereas function of dopamine D2 autoreceptor changes in opposite direction leading to a reduced [^3^H]dopamine release into the synaptic cleft. Thus, the decrease in TAAR1 activity, when it is in heterodimer form with dopamine D2 receptors, may represent a negative feedback regulation to TAAR1 signaling. Such crosstalk between TAAR1 and presynaptic dopamine D2 receptors may occur in dopaminergic-GABAergic synapses of the direct and indirect striatonigral efferent pathways.

Translocation of the intracellular TAAR1 by agonist drugs into the plasma membrane and its heterodimer formation with postsynaptic dopamine D2 receptors may also occur in GABAergic projection neurons of the striatum. Formation of TAAR1-dopamine D2 receptor heterodimers will then result in a decreased TAAR1 signaling in GABAergic neurons as well. Interaction of the postsynaptically expressed TAAR1 and dopamine D2 receptors may lead to an inhibition of GABA release causing a decline of the concentration-effect curve of (-)BPAP in postsynaptic neural elements. The site of heterodimerization of postsynaptic TAAR1 and D2 receptors may be the spines of striatopallidal projection neurons, where these receptors are located.

We also hypothetize that increased presynaptic dopamine D2 autoreceptor function, that develops following receptor heterodimerization, results in a decreased amount of dopamine released into the synaptic cleft disinhibiting GABAergic neurons from the inhibitory dopaminergic tone. This disinhibition may affect a subset of postsynaptic D2 receptors not involved in receptor heterodimerization. As a result of disinhibition, reciprocal changes in [^3^H]dopamine and [^3^H]GABA release occur: the decreased dopamine release will reduce the activity of the inhibitory postsynaptic D2 receptors and GABA release increases.

### Regulation of Pre- and Postsynaptic TAAR1: The Role of D Cells: Physiologic Considerations

Increasing number of publications concluded that the source of trace amines (phenylethylamine, tyramine, tryptamine), endogenous ligands for TAAR1, is the D cells [45]. D cells are a subset of small aspiny GABAergic interneurons in the striatum and are sensitive to kainate administration but insensitive to 6-hydroxydopamine treatment [46, 47].

It is conclusive that trace amines are not stored in vesicles in D cells; their efflux into the extrasynaptic space is due to passive diffusion across the cell membranes [48]. These compounds are highly lipophilic, and their transport processes may relate to their lipophilicity. Transporter contribution to uptake of trace amines was also not identified in striatal dopaminergic or GABAergic neurons and thus, trace amines may reach intracellularly located TAAR1 via entering these neural structures by passive diffusion [49].

D cells are aromatic acid decarboxylase (AADC, EC 4.1.1.28) immunopositive neurons expressing different isoforms of AADC but not amino acid hydroxylases, key enzymes of the syntheses of catechol- and indolamines. In trace amine synthesis, phenylalanine is decarboxylated by phenylalanine decarboxylase (EC 4.1.1.53) producing phenylethylamine and tyrosine is decarboxylated by tyrosine decarboxylase (EC 4.1.1.25) producing tyramine. Whereas trace amines are synthesized in D cells, their metabolism by monoamine oxidase occurs in presynaptic nerve endings or postsynaptic neurons [16, 48].

**Fig. 5:**
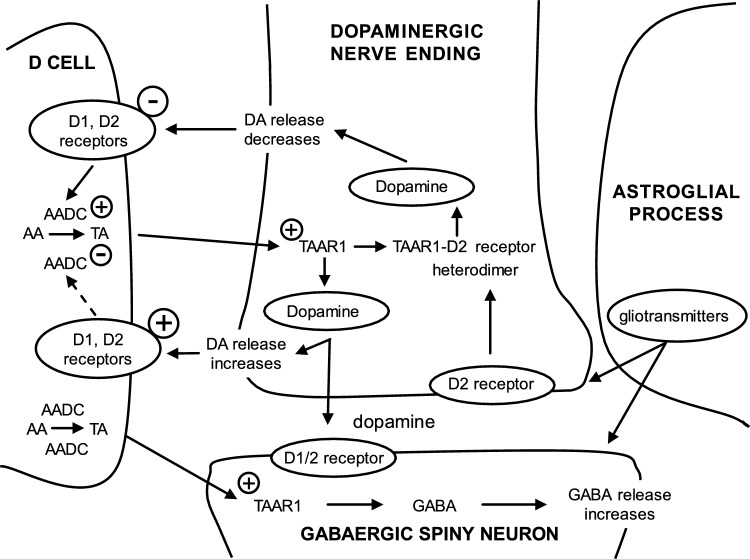
Hypothetical model for the regulation of TAAR1 and signaling. This regulation is based upon a quadripartite model with cross-talk between trace amine-producing D cells, TAAR1-expressing dopaminergic nerve endings, GABAergic neurons, and astroglial processes [22, 53]. Astroglial influences to dopaminergic axon terminals by gliotransmitters have been discussed in the striatum in details by Adermark and coworkers [54]. In our model, TAAR1 activation switches on, whereas the heterodimer form of TAAR1 with dopamine D2 receptor switches off action potential-mediated dopamine release [55]. Dopamine released into the extrasynaptic space up- or downregulates AADC activity in D cells and trace amine synthesis, which then will influence TAAR1 activity in positive or negative direction. Supporting this, recent experiments in our laboratory indicated that the trace amine phenylethylamine evokes [^3^H]dopamine release from rat striatum [17]. We also postulate here a receptor resembling to TAAR1 function in the regulation of GABA release. Abbreviations: AA, amino acid; TA, trace amine; DA, dopamine; AADC, aromatic amino acid decarboxylase; TAAR1, trace amine-associated receptor 1

There is a reciprocal relationship between dopaminergic tone and trace amine synthesis rate [48]. In the striatum, D cells presumably express dopamine D1 and D2 receptors in their cell membranes, which regulate the enzyme AADC: D1 and D2 receptor agonists decrease AADC activity, whereas antagonists exert the opposite effect [50–52]. When dopamine is released by increased TAAR1 signaling, it activates D1 and D2 receptors in D cell membranes, which then leads to decrease the activity of AADC. This enzyme activity is rate limiting in trace amine synthesis determining conversion of the corresponding amino acids to trace amines and their accumulation in D cells [48]. The reduced amount of trace amines that diffuse to TAAR1 in pre- and postsynaptic neural elements decrease the activity of TAAR1 and signaling and consequently the release of dopamine or GABA. Thus, in our model, D cells and trace amines regulate TAAR1 activity and the evoked dopamine release, establishing a regulation based on negative feedback (Fig. 5).

## Conclusion

Although a substantial knowledge has been accumulated on TAAR1 and its signaling in the last two decades, much less is known about the regulatory mechanisms involved in this process. We have proposed here a potential mechanism that may control TAAR1 operation. This negative feedback regulation is based upon increase and decrease of dopamine release evoked by TAAR1 activation or TAAR1-dopamine D2 receptor heterodimerization, respectively. Changes in dopamine release will activate or inhibit dopamine receptors (D1 and D2) expressed in D cell plasma membranes, which will then reciprocally alter the synthesis and efflux rate of trace amines, the endogenous ligands of TAAR1. To have an insight into this regulation, we used the dopaminergic activity enhancer drug (-)BPAP that can be considered as an exogenous agonist ligand for TAAR1. The neural base of this regulation is a hypothetical quadripartite model formed by pre- and postsynaptic neural structures, D cells, the source of trace amines, and supportive astroglial cell processes.

## Data Availability

No datasets were generated or analysed during the current study.

## Abbreviations

AA: Amino acid
AADC: Aromatic L-amino acid decarboxylase
(-)BPAP: (*R*)-(-)-1-(Benzofuran-2-yl)-2-propylaminopentane
CAE: Catecholaminergic activity enhancer
GABA: γ-Aminobutyric acid
MAO: Monoamine oxidase
PKA: Protein kinase A
PKC: Protein kinase C
EPPTB: RO5212773, N-(3-ethoxyphenyl)-4-pyrrolidin-1-yl-trifluoromethylbenzamide
TA: Trace amine
TAAR1: Trace amine-associated receptor 1
